# Literary Notes

**Published:** 1906-03

**Authors:** 


### LITERARY NOTES.

The British Medical Journal appeared in a new cover of bluish gray tint, at the beginning of this year, which gives this veteran an appearance of youth.

THE AMERICAN JOURNAL OF THE MEDICAL SCIENCES inagurated the current calendar year by getting into a yellow outside. It is, however, by no means a "Yellow Journal;" on the contrary it remains at present as it has been from the first, the foremost periodical exponent of American Medicine.

---

THE *Maritime Medical News,* published at Halifax, presented itself in a new dress, with double columns and an artistic cover, at the beginning of the current year. These indications of prosperity are very welcome to the contemporaries of the News on this side of the Anglo-American boundary line.

---

P. BLAKISTON'S SON & COMPANY, the publishers of Gould's medical dictionaries, announce that they sold during 1905, 17,084 copies; and that previously they had sold 181,173 copies, making a total of 198,257. This is a phenominal sale, due no doubt to the intristic merits of the books.

---

"LE JOURNAL LE PLUS CHER DU MONDE,"—the most expensive journal in the world,—in its issue for the period embraced between the fifteenth of August and the first of September 1905, gave an account of the gondola dinner given by Mr. George A. Kessler, in the court of the Savoy Hotel, London, June 30, 1905. Alfred Harvey wrote the description and said the court was canopied to imitate a Venetian sky, and was flooded with water to permit the huge gondola to float. It contained 24 persons and the feast and decorations cost $15,000. Mr. Kessler, it will be remembered, is the head of the firm of George A. Kessler and Company, importers and distributors of the celebrated Moet & Chandon champagne. The vintage of 1900 now being marketed is the choicest wine in the world, the "white seal" being an especial favorite. A double-page picture of the gondola dinner adorns this number of "Le Journal."

